# UV-C and UV-C/H₂O-Induced Abiotic Degradation of Films of Commercial PBAT/TPS Blends

**DOI:** 10.3390/polym17091173

**Published:** 2025-04-25

**Authors:** K. Gutiérrez-Silva, Antonio J. Capezza, O. Gil-Castell, J. D. Badia-Valiente

**Affiliations:** 1 Research Group in Materials Technology and Sustainability (MATS), Department of Chemical Engineering, School of Engineering, University of Valencia, Av. Universitat s/n, 46100 Burjassot, Spain; karen.gutierrez@uv.es; 2 Fibre and Polymer Technology Department, KTH Royal Institute of Technology, Teknikringen 56, SE-100 44 Stockholm, Sweden; ajcv@kth.se

**Keywords:** abiotic degradation, ultraviolet C (UV-C) irradiation, bioplastic, poly(butylene adipate-co-terephthalate (PBAT), thermoplastic starch (TPS)

## Abstract

The environmental impact of conventional plastics has spurred interest in biopolymers as sustainable alternatives, yet their performance under abiotic degradation conditions still remain unclear. This study investigated the effects of ultraviolet C (UV-C) irradiation and its combination with water immersion (UV-C/H_2_O) on films of commercial poly(butylene adipate-*co*-terephthalate)-thermoplastic starch (PBAT/TPS) blends. Changes in structural, chemical, morphological, and thermal properties, as well as molar mass, were analyzed. The results showed distinct degradation mechanisms during exposure to UV-C irradiation either in dry or during water-immersion conditions. UV-C irradiation disrupted PBAT ester linkages, inducing photodegradation and chain scission, leading to a more pronounced molar mass decrease compared to that under water immersion, where a more restrained impact on the molar mass was ascribed to diffuse attenuation coefficient of irradiation. Nevertheless, under UV-C/H_2_O conditions, erosion and disintegration were enhanced by dissolving and leaching of mainly the TPS fraction, creating a porous structure that facilitated the degradation of the film. Blends with higher TPS content exhibited greater susceptibility, with pronounced reductions in PBAT molar mass. In conclusion, exposure of films of PBAT/TPS blends to ultraviolet/water-assisted environments effectively initiated abiotic degradation, in which fragmentation was accentuated by the contribution of water immersion.

## 1. Introduction

Polymers are widely used across various industries due to their versatility and functionality. However, the environmental persistence of conventional plastics has raised global concerns, driving increased interest in biodegradable bioplastics as sustainable alternatives, regardless of their renewable or non-renewable origin [1].

Bioplastics currently account for approximately 0.5% of the total global plastic production, which exceeds 400 million tons annually [2]. After a period of stagnation, overall bioplastic production rose again in 2024 up to around 2.47 million tons, driven by increasing demand and the development of more advanced applications and products, and projections estimate that this will rise to 5.73 million tons by 2029 [3]. This growth is fueled by heightened environmental awareness, regulatory pressures, and innovations in materials science, which enable bioplastics to be utilized in a wider range of sectors, including packaging, agriculture, medicine, and other consumer goods [4,5,6,7,8].

Among biodegradable bioplastics, poly(butylene adipate-co-terephthalate) (PBAT) stands out as a promising material due to its fully biodegradable nature and its favorable mechanical properties, which rival those of conventional commodity plastics [9]. Unlike many other biodegradable polyesters, PBAT offers flexibility and toughness, making it suitable for a range of applications such as packaging and agricultural films [10,11,12]. However, despite these advantages, the high production cost and restricted biodegradability of PBAT limits its widespread adoption [13]. To address this challenge, blending PBAT with other bioplastic materials, such as thermoplastic starch (TPS), has been explored as a strategy to reduce costs, tailor its performance, and enhance its biodegradability. The development of PBAT/TPS blends not only improves the economic feasibility of PBAT but also provides an opportunity to develop fully bio-degradable plastic materials with tunable physicochemical behavior [14,15,16,17,18,19,20,21].

Like traditional plastics, bioplastics also become waste at the end-of-life stage. Currently, efforts to manage this waste focus on advanced recycling methods and valorization strategies to enhance environmental performance, and reduce landfill disposal [22]. However, a better understanding of bioplastics’ end-of-life performance is needed, as multiple mechanisms influenced by external factors can operate simultaneously to promote degradation, with potential synergistic performances [23].

In addition to the natural alteration that bioplastics undergo when exposed to environmental conditions, it is widely known that applying advanced accelerated degradation techniques can significantly reduce ulterior degradation times [24]. Methods such as thermal degradation, photolysis, chemical degradation, plasma treatment, and technologies that induce high amounts of energy, such as microwave irradiation, ultrasonication, etc., have been demonstrated to be effective in modifying bioplastics when facilitating their handling at the end-of-life stage [25]. In this regard, abiotic degradation mechanisms include mechanical, thermal/thermo-oxidative, photo/photo-oxidative, and chemical (principally hydrolysis or oxidation), which targets molecular-level disintegration and degradation [26,27,28,29]. In particular, the application of degrading agents in the presence of oxygen-inducing oxidative degrading reactions has led to Advanced Oxidation Processes (AOPs), which gained interest in recent years to serve as a pre-treatment strategy for polymeric materials at the end-of-life stage [30,31].

Among abiotic degradation approaches, UV radiation exposure plays a critical role in the degradation of bioplastics [26,30,32,33]. In general, the mechanisms of photodegradation involve the transfer of UV energy to the polymer, which disrupts its structural stability, leading to photoionization, oxidation, and chain scission [34,35]. Typical photodegradation reactions, such as Norrish Type I and Norrish Type II, involve the generation of free radicals [36,37], which ultimately promote the fragmentation of polymer chains [32,38]. In the presence of oxygen, UV irradiation facilitates the formation of reactive oxygen species that cause photo-oxidative degradation [39]. PBAT, in particular, is highly susceptible to photodegradation due to the presence of carbonyl groups in its molecular structure [40]. These groups act as photosensitizers, enhancing the absorption of UV radiation and accelerating the degradation process. Given PBAT’s inherent sensitivity to UV light, particularly in the UV-C range (100–280 nm), photodegradation offers a viable approach for accelerating the breakdown of PBAT-based materials in controlled environments [41]. Also, in the case of polyesters like PBAT, photodegradation and hydrolytic degradation often occur simultaneously, particularly in environmental conditions that combine UV exposure with moisture [42]. By altering the surface properties of the plastic and reducing the molecular weight of the polymer chains, UV exposure can make the material more susceptible to enzymatic [43] or microbial degradation [36,44,45]. This dual approach—combining abiotic degradation with biotic processes—provides an effective strategy for managing the lifecycle of biopolymers like PBAT-based materials. Table 1 gathers some valorization strategies reported to accelerate the biodegradation of biopolymers in various environments.

Previous studies have demonstrated the consequences of different environmental factors, such as light, water, and temperature, on the degradation of PBAT-based blends. For instance, the degradation of PLA/PBAT-based films was promoted by the action of dissimilar environmental factors, with hydrolysis and photolysis acting as the main degradation mechanisms [34,35,36]. Moreover, PBAT/PLA mulch films subjected to indoor UV-accelerated degradation were compared with field cultivation environment degradation to generate a predictive model to estimate long-term service life [11]. Moreover, the degradation of PLA/PBAT films was evaluated under the action of dissimilar environmental factors, with hydrolysis and photolysis acting as the main degradation mechanisms [41,42,47]. The effect on color, mechanical, and thermal properties were key indicators of PBAT film degradation under environmental conditions. Additionally, studies with PBAT/TPS blends have shown that the biodegradation rate and compostability increase with the addition of plasticized starch, further improving the overall biodegradability of the material [48,49]. However, there is a lack of research on when PBAT/TPS blends are exposed to UV-C radiation during water immersion, for accelerating the abiotic degradation of the material. Therefore, understanding the consequences of this combined approach is important to gain knowledge on how different environmental factors interact to degrade promising biopolymeric materials such as PBAT/TPS blends.

Altogether, the present study aims to investigate the effects of the simulated and controlled abiotic treatment of films of commercial PBAT/TPS blends with UV-C irradiation both in dry conditions and during water immersion. By targeting the UV-C region of the spectrum, the most energetic wavelengths can be applied to accelerate the degradation process of the biopolymeric blends within a controlled timeframe. Following this accelerated aging, the characterization of surface and bulk properties of the materials is crucial, with specific emphasis on the correlation among structure, morphology, and properties after exposure. By understanding how UV-C radiation in combination with water affects the physicochemical properties of PBAT/TPS commercial blends, this research may elucidate how UV-C irradiation can be applied in organized waste management systems as a potential strategy to promote accelerated degradation of biopolymers.

## 2. Materials and Methods

### 2.1. Materials

Commercial blends of PBAT/TPS were supplied by Prime Biopolymers (Paterna, Spain) under the grade ZIMIA for flexible packaging, and processed into 100 µm thick films by the Institute of Technology of Plastics, AIMPLAS (Paterna, Spain). The materials, with different percentages of components in their structure, were labeled as PT1, PT2, and PT3, with increasing mass proportion of TPS, according to results shown in Section 3.1. Even though the particular nature of the additives was not indicated, manufacturers attribute them to plasticizing and coupling features. HPLC-grade chloroform was provided by Sigma-Aldrich (St. Gallen, Switzerland).

### 2.2. Ultraviolet C Irradiation

Films were exposed to ultraviolet C irradiation (UV-C) in a Vilber Lourmat CN-15 dark chamber (Marne-la-Vallée, France) equipped with four lamps with a total irradiance of 1.75 mW·cm^−2^ and lamp power of 15 W at 254 nm. The films were cut into dimensions of 25 × 25 mm^2^ and were exposed to the UV-C source for 24, 48, 72, and 96 h at a distance of 5 cm, and a temperature of 40 ± 2 °C. Under these conditions, the UV dose was approximated for the different times, being 151.2, 302.4, 453.6, and 604.8 J·cm^−2^, respectively. Exposure to UV-C in combination with water was carried out during immersion using Petri dishes of 7 cm diameter filled with 20 mL of distilled water, with the sample immersed at about 5 mm, and with the temperature for the water bath maintained at 35 ± 2 °C. After each extraction, the immersed samples were dried with a tissue to remove the water from the surface and dried under vacuum at 25 °C until constant weight to remove remnant humidity. Then, all of them were introduced into zip bags and stored for further analysis at normalized lab conditions [50].

### 2.3. Characterization Methods

#### 2.3.1. Colorimetry

The color change was determined with a StellarNet colorimeter (Tampa, FL, USA) consisting of a BLACK-Comet-CXR-100 spectrometer, an SL1-Filter tungsten halogen lamp, an R600-8-VisNIR reflectance probe, an RS50 white reflectance standard, an RPH probe holder, and SpectraWiz Spectroscopy Software v6.3 using the CIELAB system. In this system, the color is expressed in terms of L*, a*, and b* color coordinates, where the L* represents lightness, moving from white (100) to black (0), a* indicates the change between red (+) and green (-), and b* the shift from yellow (+) to blue (-). Parameters were measured against the white reflectance standard and were directly obtained from the colorimeter. To assess the possible variability, three measurements were made at different locations on the irradiated films. The change in color ΔE was calculated according to Equation (1).

(1) $$∆E=\sqrt{{\left(∆a^{*}\right)}^{2}+{\left(∆b^{*}\right)}^{2}+{\left(∆L^{*}\right)}^{2}}$$

where Δ$a^{*}$, Δ$b^{*}$, and Δ$L^{*}$, respectively, represent the change in the color coordinates compared to the sample before UV treatment. 

#### 2.3.2. Field-Emission Scanning Electron Microscopy (FE-SEM)

The surface of the specimens was analyzed through a Hitachi S-4800 field emission scanning electron microscope (FE-SEM) (Tokyo, Japan). The samples were mounted on metal studs and Au/Pd sputtered under vacuum conditions for 90 s using a Cressington 208HR high-resolution sputter coater (Watford, UK), equipped with a Cressington thickness monitor controller. The working conditions in FE-SEM were a voltage of 5 kV and a working distance of 8 mm.

#### 2.3.3. Water Contact Angle (WCA)

A custom-made assembly was used to measure the water contact angle. Briefly, measurements were carried out on a flat base at which a Celestron 44301 micro camera (Torrance, CA, USA) was pointed, with a Philips Hue White Ambiance spotlight (Eindhoven, The Netherlands), a KF Technology NE300 peristaltic pump (Rome, Italy) that drove at a flow rate of 10 µL·min^−1^ to generate the drop of 2.5 µL, and a 0.1 mm diameter needle, through which the drop fell onto the flat surface. For consistency purposes, the contact angle was measured after 15 s of drop deposition in all cases. Images were processed with the ImageJ software v1.54g (Bethesda, MD, USA).

#### 2.3.4. Fourier-Transform Infrared Spectroscopy (FTIR)

Fourier-transform infrared spectroscopy (FT-IR) was used in the attenuated total reflectance (ATR) mode. Spectra were obtained in the wavenumber range of 4000 to 500 cm^−1^, with a resolution of 4 cm^−1^, from 32 scans on an Agilent Technologies Cary 630 FTIR Spectrometer (Santa Clara, CA, USA). Results were evaluated considering the specific IR bands at 3470 cm^−1^, related to the stretching of hydroxyl groups; at 1710 cm^−1^, associated with the C=O stretching of carboxylic acids; at 1018 cm^−1^, ascribed to the glycosidic bonds of starch molecules; and at 727 cm^−1^, ascribed to the out-of-plane bending vibration of the C–H in the phenylene ring in PBAT [51,52,53]. The variations in these bands relative to the pristine materials were considered as degradation indicators and were calculated using Equations (2), (3), (4) and (5), giving as a result the indexes Δ*I*_3420_, Δ*I*_1710_, Δ*I*_1018_, and Δ*I*_727_, respectively.

(2) $${∆I}_{3420}(\%)=\frac{I_{3420\;treated}-I_{3420\;pristine}}{{∆I}_{3420\;pristine}}\times 100$$

(3) $${∆I}_{1710}(\%)=\frac{I_{1710\;treated}-I_{1710\;pristine}}{{∆I}_{1710\;pristine}}\times 100$$

(4) $${∆I}_{1018}(\%)=\frac{I_{1017\;treated}-I_{1017\;pristine}}{{∆I}_{1017\;pristine}}\times 100$$

(5) $${∆I}_{727}(\%)=\frac{I_{727\;treated}-I_{727\;pristine}}{{∆I}_{727\;pristine}}\times 100$$

#### 2.3.5. Thermogravimetric Analysis (TGA)

Thermal stability was assessed using a TA Instruments TGA 550 device (New Castle, DE, USA). Samples with a mass of around 3 mg were introduced in platinum crucibles and were measured from 30 to 900 °C with a heating rate of 10 °C·min^−1^ under an inert atmosphere of N_2_ at a flow rate of 50 mL·min^−1^. The specimens were characterized in triplicates and the averages of mass loss and temperatures were taken as representative values.

#### 2.3.6. Differential Scanning Calorimetry (DSC)

Calorimetric data were obtained by differential scanning calorimetry (DSC) using a Setaram Setline+ DSC (Caluire-et-Cuire, France). The samples, with a mass of around 3 mg, were placed in 30 μL aluminum crucibles. Thermal properties were assessed over heating/cooling segments of 10 °C·min^−1^ between 40 and 180 °C. All experiments were performed under an N_2_ atmosphere at a flow rate of 50 mL·min^−1^. The specimens were characterized in triplicates and the averages of temperatures and enthalpies were taken as representative values.

The lamellar thickness of the PBAT crystalline population was calculated by applying the Thomson-Gibbs equation (Equation (6)), based on the temperatures associated with the melting transitions [54,55],

(6) $$l_{C}\left(T_{m}\right)={\left[\left(1-\frac{T_{m}}{T_{m}^{0}}\right)\cdot \frac{{∆h}_{mV}}{2\cdot \sigma_{e}}\right]}^{-1}$$

where *T_m_* is the melting peak temperature; *T_m_^0^* is the equilibrium melting temperature of an infinite crystal (from 411 to 451 K, according to the adipate/terephthalate content) [56]; *σ_e_* is the surface free energy of the basal plane where the chains fold (75 × 10^−3^ J·m^−2^) [57,58]; and *∆h_mV_* is the melting enthalpy per volume unit (1.43 × 10^8^ J·m^−3^).

#### 2.3.7. Gel Permeation Chromatography (GPC)

Gel permeation chromatography analyses were carried out using a Malvern Instruments GPCMAX chromatograph (Worcestershire, UK), containing a PLgel 5 µm guard column (7.5×) and two Agilent PLgel 5 µm MIXED-D (300 × 7.5 mm) columns (Santa Clara, CA, USA). The samples were dissolved in chloroform with concentrations of around 2.0 mg·ml^−1^ and filtered through 0.45 µm PTFE filters. As well, chloroform was used as the mobile phase at a flow rate of 1 mL·min^−1^ and the column temperature was set at 35 °C. Monodisperse polystyrene standards with a narrow dispersity were used for calibration (162–364,000 g·mol^−1^). The specimens were characterized in duplicates and the averages of molar mass results were taken as representative values.

## 3. Results and Discussions

As a result of the UV-C irradiation and water immersion exposure, a series of changes were induced at both microscopic and macroscopic levels. Therefore, alterations in appearance, morphology, composition, thermal properties, stability, and molar mass of PBAT/TPS films were ascertained.

### 3.1. Initial Physico-Chemical Properties of Films of Commercial PBAT/TPS Blends

**Figure 1:**
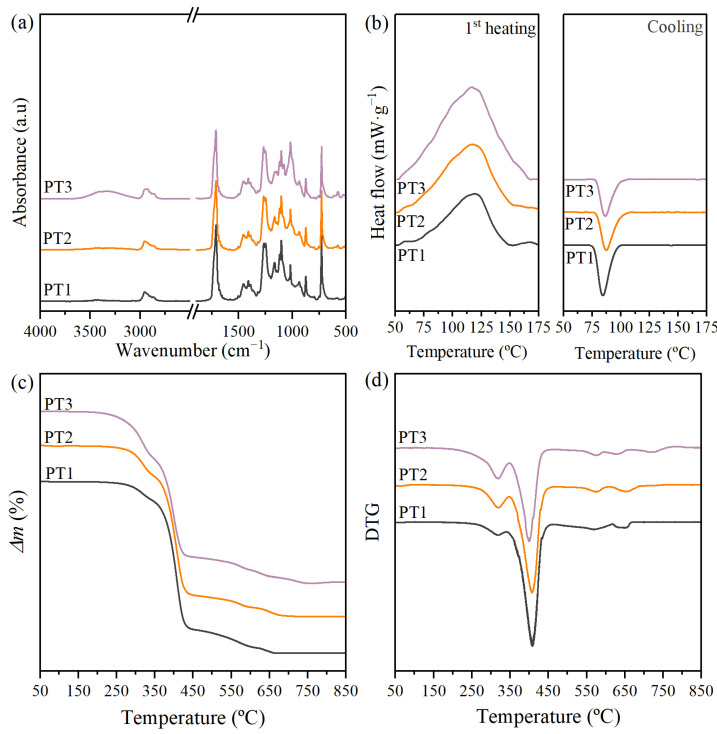
(**a**) FTIR spectra, (**b**) calorimetric thermograms for the first heating and cooling segments, (**c**,**d**) thermogravimetric and derivative thermogravimetric curves of PBAT/TPS commercial blends.

The films of PBAT/TPS commercial blends were preliminarily assessed, to set the baseline for further characterizations. Table 2 gathers some of the most representative properties, including the film’s thickness, macroscopic appearance, color parameters, microscopic surface, water contact angle, molar mass, and thermal properties. The infrared spectra to evaluate composition as well as calorimetric and thermogravimetric thermograms are shown in Figure 1.

In general, the samples showed a smooth morphology with a whitish hue and partial translucency. The transparency gradually decreased with the addition of TPS. The color characterization was assessed through the evaluation of greening (a*), yellowing (b*), and lightness (L*) indexes. The most relevant observation was the color change towards a more yellow feature together with a lower lightness with the increased percentage of TPS. This change has been ascribed to the particular color of starch together with the possibility of the loss of structure and crystallinity of starch granules during processing, which may promote color shifts towards browning [59].

Surface electron micrographs revealed a heterogeneous microstructure with two distinct phases that could be identified in all samples. The granules present on the surface are indicative of discrete starch domains dispersed within the PBAT matrix, highlighting phase separation and heterogeneity. This is correlated with the chemical disparity of the components due to differences in polarity and molecular structure. According to the reported performance in PBAT/TPS blends, the continuous phase is formed by the PBAT polymer, while the dispersed phase is attributed to TPS [60]. Blending hydrophobic PBAT with hydrophilic starch may present challenges of immiscibility and incompatibility [61,62,63], though an increase in TPS content improves the dispersion homogeneity of the TPS/PBAT blends [63]. Rather than leading to a decrease in the number of dispersed particles, the increase in TPS content significantly reduced the size of the starch granules within the blends. This phenomenon is consistent with the literature, indicating that blends of PBAT and TPS with TPS contents greater than 20–30% have a dispersed phase of TPS droplets inside of a continuous matrix of PBAT [59,63,64]. Also, the amount and nature of compatibilizers and plasticizers can determine the size of TPS particles [65].

Furthermore, water contact angle measurements showed a hydrophilic behavior in all cases with WCA below 90°. As expected, films with higher PBAT content present a lower wettability and enhanced hydrophobicity, while a higher TPS proportion results in greater hydrophilicity [14]. PBAT, a co-polyester consisting of both aliphatic and aromatic segments, is well-known for its high resistance to water interaction [62]. On the other hand, TPS has a strong affinity for water due to the abundance of hydroxyl groups provided by both starch and plasticizers [62,66]. These hydroxyl groups in the starch structure easily bond with water molecules, forming hydrogen bonds that enhance moisture absorption and increase the film’s wettability [14,64,67,68].

The infrared spectra of pristine films are presented in Figure 1a. PBAT typically presents bands around 1268 cm^−1^ and 1709 cm^−1^ which correspond to C–O and C=O vibrations of the ester groups in the backbone [14,69,70]. The main absorption bands of the TPS were observed in all the spectra and were especially relevant for the composition with greater TPS content. A broad absorption peak ranging from 3000 to 3600 cm^−1^, which is centered at 3350 cm^−1^, was ascribed to O-H stretching vibration driven by intra and inter-molecular hydrogen bonding due to hydroxyl groups [14,15,71]. A progressive increase of this band with a shift towards lower wavenumbers was observed as the TPS presence increased [14].

In the calorimetric thermograms for the first heating scan plotted in Figure 1b, a broad endothermic transition from 40 to 160 °C was observed corresponding to the melting of the PBAT crystalline phase, with a minor process around 60 °C and a more prominent peak around 120 °C. Due to PBAT being a block copolymer, it may exhibit dissimilar melting transitions associated with crystalline domains given by its comonomers. The butylene adipate (BA) segment is called the soft segment because of its aliphatic structure, and the butylene terephthalate (BT) segment is the rigid segment due to the aromatic terephthalate [71]. Some studies in the literature ascribed the first endothermic peak to the melting of a crystal lattice containing mainly BA units, whereas the second one was correlated to the fusion of crystals related to the stiffer BT segment [68]. The existence of various types and sizes of crystals due to the BA and BT segments together with the possible interactions with TPS domains and the effect of other components in the mixture may explain the observed PBAT’s broad melting transition. The melting enthalpy (Δ*h_m_*) remained virtually constant for PT1 and PT2 with values of around 30 and 29 J·g^−1^, whereas a significant increase up to 46 J·g^−1^ was observed as the TPS content increased for PT3. The melting temperature (*T_m_*) remained practically unaltered in the vicinities of 120 °C. The apparent crystallinity degree (*X_c-app_*) of the PBAT phase was calculated as 32%, 34%, and 50% for PT1, PT2, and PT3, respectively.

The mass loss and derivative thermogravimetric curves are shown in Figure 1c and Figure 1d, respectively, and were used to evaluate the thermal stability of pristine films and estimate the composition in terms of PBAT and TPS percentages. It was found that the thermal degradation process occurred in a multi-stage process. In the early stages, the mass loss at around 150 °C was related to moisture and plasticizer loss. The first significant degradation step may be associated with the TPS phase, occurring with a maximum rate at around 320 °C, which falls within the range of 160–380 °C, observed as a minor peak in the DTG curve [72]. Then the second and more significant peak around 400 °C is due to the PBAT thermal decomposition [48,71], followed by the decomposition of char. A gradual rise in the mass loss percentage near 300 °C was observed, following the sequence of increasing TPS content [67]. The estimated mass contribution for this stage correlated to the TPS phase was around 10, 17, and 26%, which are typical percentages for the preparation of PBAT/TPS blends [61,62,63]. As expected, the onset temperature decreased with more TPS content, due to its lower thermal stability [48]. Lastly, it is worth noting that beyond 600 °C, where char decomposition occurred, a residual mass of about 4–5% was found, likely due to the presence of inorganic fillers in the composition.

Finally, the molar mass of the PBAT fraction was evaluated in terms of the average molar mass in number (*M_n_*) and weight (*M_w_*), as well as the polydispersity index (*PDI*). The obtained values for *M_n_* were in the range from 55 × 10^3^ to 63 × 10^3^ g·mol^−1^ and *M_w_* from 113 × 10^3^ to 120 × 10^3^ g·mol^−1^, which involved *PDI* values of around 2, typically reported before for commercial PBAT-based compositions [73]. It is relevant to highlight that the incorporation of greater percentages of TPS into the blends resulted in a slightly higher molar mass of PBAT fraction. About 5% and 7.5% higher *M_n_* was found, which can be correlated to the plasticizing performance of TPS in the PBAT matrix, which may have prevented thermo-mechanical degradation during processing.

### 3.2. Consequences of UV-C/H_2_O-Driven Abiotic Degradation

#### 3.2.1. Macroscopic Changes

The UV-C irradiation induces significant changes in the structure and properties of polymers, with color alteration being one of the most relevant consequences. When polymers are exposed to UV radiation, the photons interact with the macromolecules, causing modifications in their functional groups. These changes affect the absorption and emission of visible light, resulting in discoloration [74], yellowing [10], or changes in the gloss [37,74].

**Figure 2:**
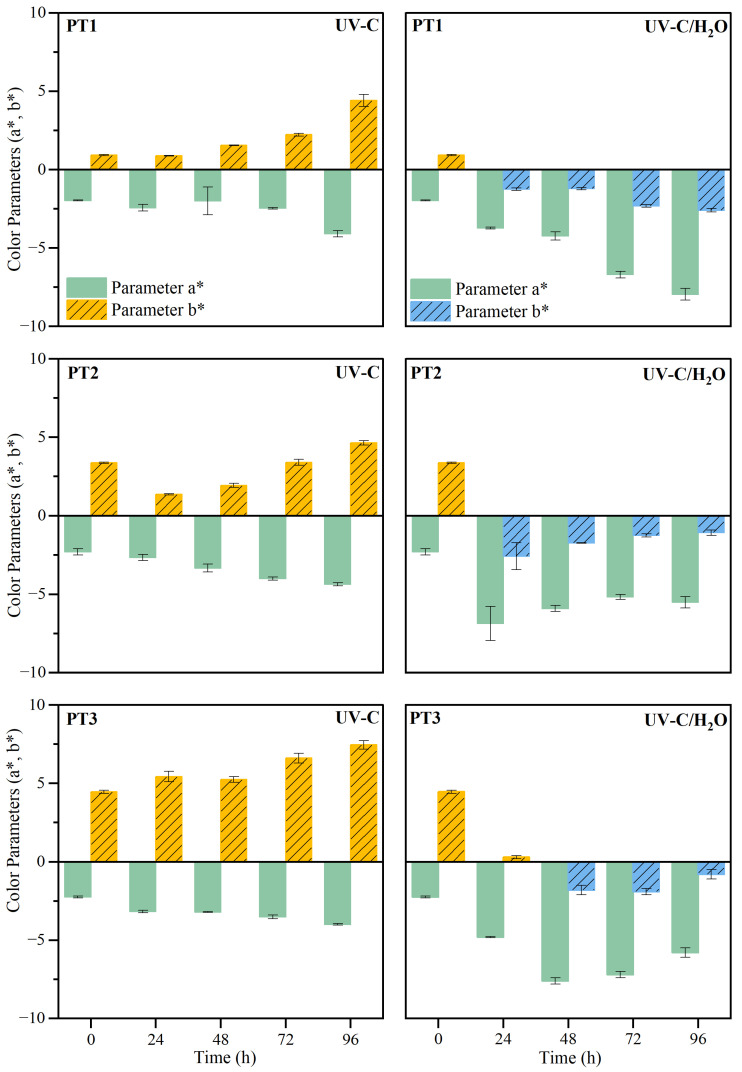
Color parameters $(a^{*},\;b^{*})\;$ for PT1, PT2, and PT3 films after UV-C irradiation in dry (UV-C) and during immersion (UV-C/H_2_O) conditions.

The macroscopic changes following both direct UV-C irradiation and the combination of irradiation with water immersion are qualitatively presented in Figure S1 of the Supplementary Materials in terms of surface photographs. After irradiation exposure, no significant loss of dimensional stability was observed, although changes in the optical properties were intuited, with a different pattern when irradiation was carried out during immersion. The quantitative evaluation of the observed changes was carried out through the study of the color variation [75]. In detail, the exposure to UV-C irradiation caused changes in the $a^{*}$ and $b^{*}$ indexes in the films, which tendencies are shown in Figure 2. Full-color data parameters ($L^{*}$ index) and total color change (ΔE) can be found in Table S1 of the Supplementary Materials.

Under dry conditions, exposure to UV-C resulted in a decrease in lightness ($L^{*}$), with an increase in the greening ($a^{*}$) and yellowing ($b^{*}$) tendencies in all cases. The change in color of PBAT-based materials has been previously reported in the literature, with UV radiation promoting that PBAT films become more yellow and opaque [10]. A progressive increase of color change (ΔE) was noted for samples with higher PBAT content (PT1 and PT2), while a peaked performance was observed after 24 h for samples with higher TPS content. The contribution of TPS during UV-C irradiation was especially visible when the yellowing index was evaluated, showing a more intense yellow color in the PT3 composition. This performance is usually accompanied by a decrease in the chain length of starch and the formation of carbonyl and carboxyl groups along the chains [76,77], which compete with crosslinking reactions after advanced exposure to UV-C irradiation [78]. Such color differences have been reported in the literature for PBAT/TPS blends, resulting in the generation of new chromophore groups that promote alterations in the color indexes [37].

In contrast, when UV-C irradiation was applied during water immersion, there was an increase in lightness ($L^{*}$) and a shift in color components, with an increase in greening ($a^{*}$) and a reversal of yellowing ($b^{*}$) indexes towards a global bluish tendency. The change from a yellow index before treatment to a blueish index from the very beginning of the aging process suggests a dissimilar performance of the film’s degradation during immersion. The color change was more pronounced after 24 h for all compositions, particularly for greater TPS percentages, followed by stabilization thereafter. This suggests that a higher proportion of TPS in the initial composition resulted in the acceleration of the greening process. As for the PBAT, the change to a bluish tendency has been reported in PBAT-based films [37], especially when degradation was caused by hydrolysis breakage of polymer chains [67]. As it has been established previously, PBAT is a polyester that may hydrolyze when it comes into contact with water, especially during immersion [79]. Therefore, chain scission is expected during immersion in the PBAT phase due to the hydrolytic breakage of ester bonds and the subsequent color change.

#### 3.2.2. Microscopic Morphology and Surface Properties

**Figure 3:**
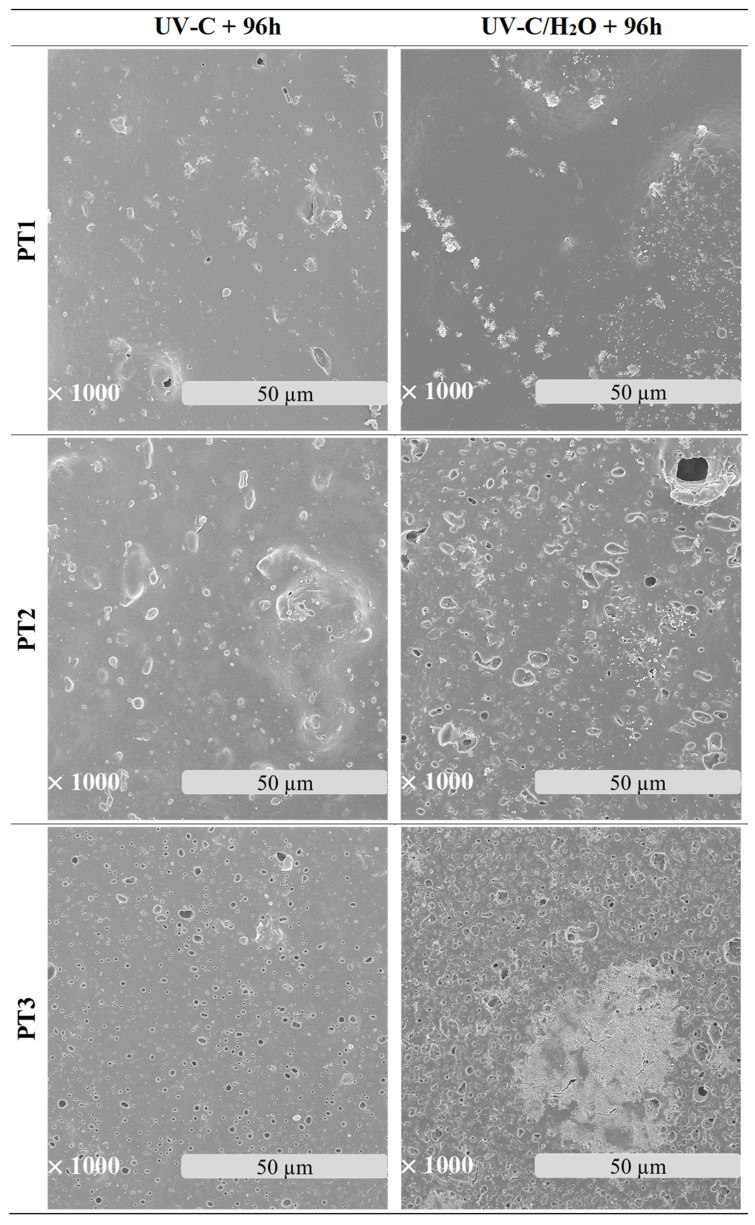
Surface microscopy images of PBAT/TPS films after 96 h of UV-C irradiation in dry (UV-C) and during immersion (UV-C/H_2_O) conditions.

The microscopic morphological changes along with the variations of the surface properties of the films were evaluated in this section. Figure 3 shows the surface electronic micrographs following 96 h of UV-C irradiation, both under dry and immersion conditions, which reveal a dissimilar pattern according to the irradiation conditions and the film composition.

Exposure to UV-C irradiation in dry conditions presumably affected the dispersed starch granules within the PBAT matrix, particularly in films with the highest TPS content. As TPS percentage increased more susceptible damage was induced, leading to the formation of dispersed pores [80]. These pores grow significantly in both size and number as degradation progresses. In line with the literature, the photodegradation of PBAT/TPS films started as a surface degradation, mainly focused on TPS domains, and was then propagated throughout the PBAT polymer matrix [39].

After UV-C irradiation of the films during water immersion, surface damage was aggravated, especially when the starch content increased [42]. In particular, the performance of water acting as a plasticizer may weaken the interactions between PBAT and TPS, which leads to the formation of microcracks and pores, altogether promoting the hydrolytic degradation in bulk and the leaching of the soluble components [60,81], such as the plasticizers and the TPS fraction. Surface deterioration became evident both in localized areas, predominantly ascribed to TPS domains, and more uniformly across the PBAT polymer matrix. The solubilization of the TPS portion may be responsible, in turn, for the observed porosity generation during the aging treatment. While hydrothermal degradation of PBAT involves the generation of holes on the surface [41,42,82], as well as microcracks [62], the generation of more fragile areas in the PBAT matrix is likely the result of indirect effects, such as hydrolysis and leaching of TPS and plasticizer, rather than a direct effect of UV-C irradiation.

It is known that water absorbs UV radiation, especially in the UV-C range (100–280 nm), which may reduce the dose received by the polymer film. Moreover, as UV light penetrates water, its intensity decreases exponentially with depth, which phenomenon is described by the diffuse attenuation coefficient [83]. According to the Lambert–Beer Law, the reduction in the transmitted irradiation is about 97.2% through a water layer of 5 mm and an absorption coefficient at 254 nm of 55.83 ± 15.65·10^−3^ m^−1^ [84].

**Figure 4:**
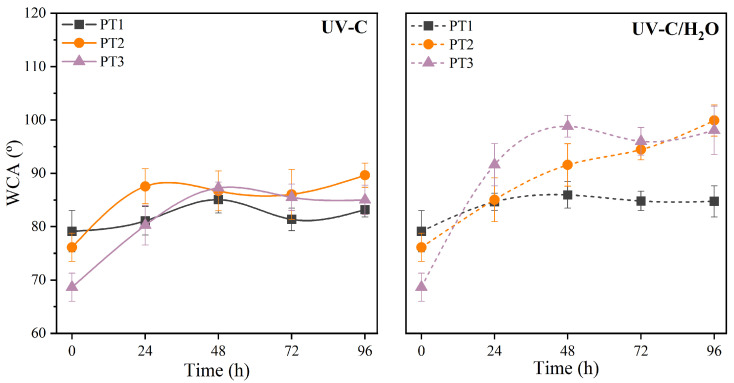
Water contact angle (WCA) of PBAT/TPS films after UV-C irradiation in dry (UV-C) and during immersion (UV-C/H_2_O) conditions. Lines are given for the sake of visual aid.

The surface properties of the films after irradiation were subsequently evaluated through the study of the water contact angle (WCA) to determine changes in the hydrophilic performance. Figure 4 shows the WCA of films of PBAT/TPS after UV-C irradiation in dry and during immersion conditions.

In films exposed to UV-C irradiation in dry conditions, a progressive increase in the contact angle was observed with prolonged exposure indicating a more hydrophobic surface. The WCA increased by 7.5% (PT1), 17.8% (PT2), and 27.1% (PT3) with an asymptotic performance above 48 h. As the UV radiation breaks down polymer chains, an increase in C-C bonds on the surface, together with a reduction of C–O or O–C=O bonds, is likely to occur, as reported for other polyesters under UV irradiation [85], making the surface more hydrophobic.

Similarly, films subjected to UV-C irradiation during water immersion also show a consistent and more pronounced increase in WCA, with rises of 8.6% (PT1), 31.3% (PT2), and 44.0% (PT3), respectively, to the initial WCA, indicating a shift toward increased hydrophobicity over time. The evaluation of the WCA suggested a change to a more hydrophobic performance the higher the initial percentage of TPS was in the blend. According to previous observations, the degradation process may have caused the leaching of hydrophilic components, particularly starch, from the PBAT/TPS blend, which in turn may result in a PBAT-rich surface inherently more hydrophobic.

#### 3.2.3. Chemical Structure

**Figure 5:**
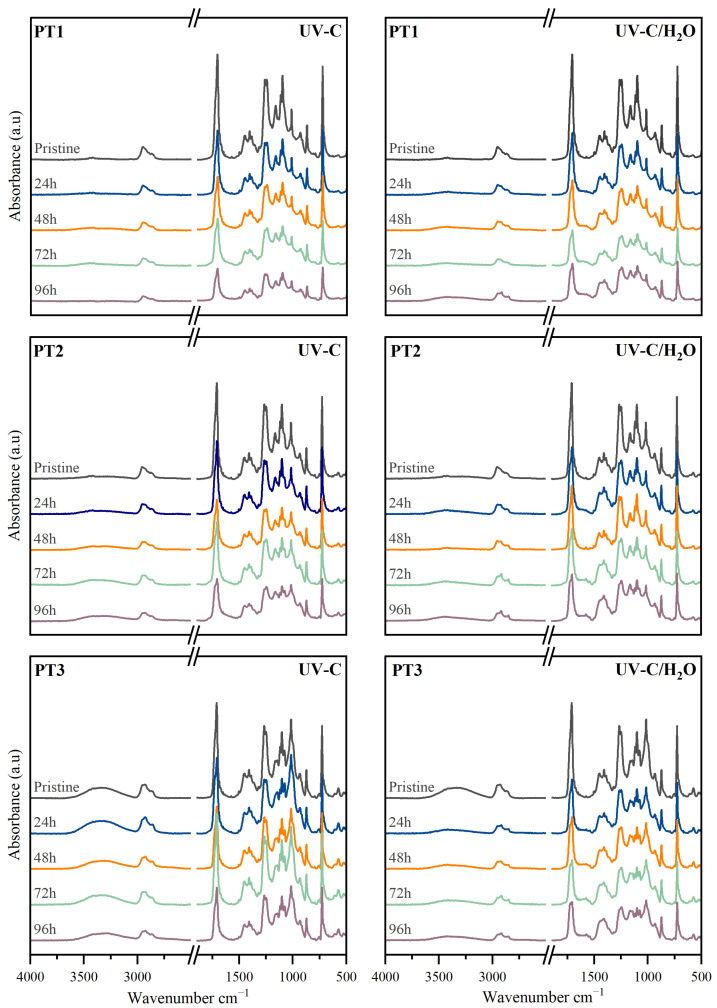
Infrared absorbance spectra of PBAT/TPS films after UV-C irradiation in dry (UV-C) and during immersion (UV-C/H_2_O) conditions.

Identifying specific functional groups and tracking changes in their concentration is essential for understanding structural modifications in polymeric materials during degradation. The appearance or disappearance of particular chemical groups offers valuable insights into reaction mechanisms and the formation of degradation products [86]. In this regard, the study of infrared spectra enables a detailed assessment of the chemical evolution of the material, providing important information regarding its stability and performance [75]. The absorbance infrared spectra of PBAT/TPS films after UV-C irradiation in dry (UV-C) and during immersion (UV-C/H_2_O) conditions were ascertained and are presented in Figure 5.

**Figure 6:**
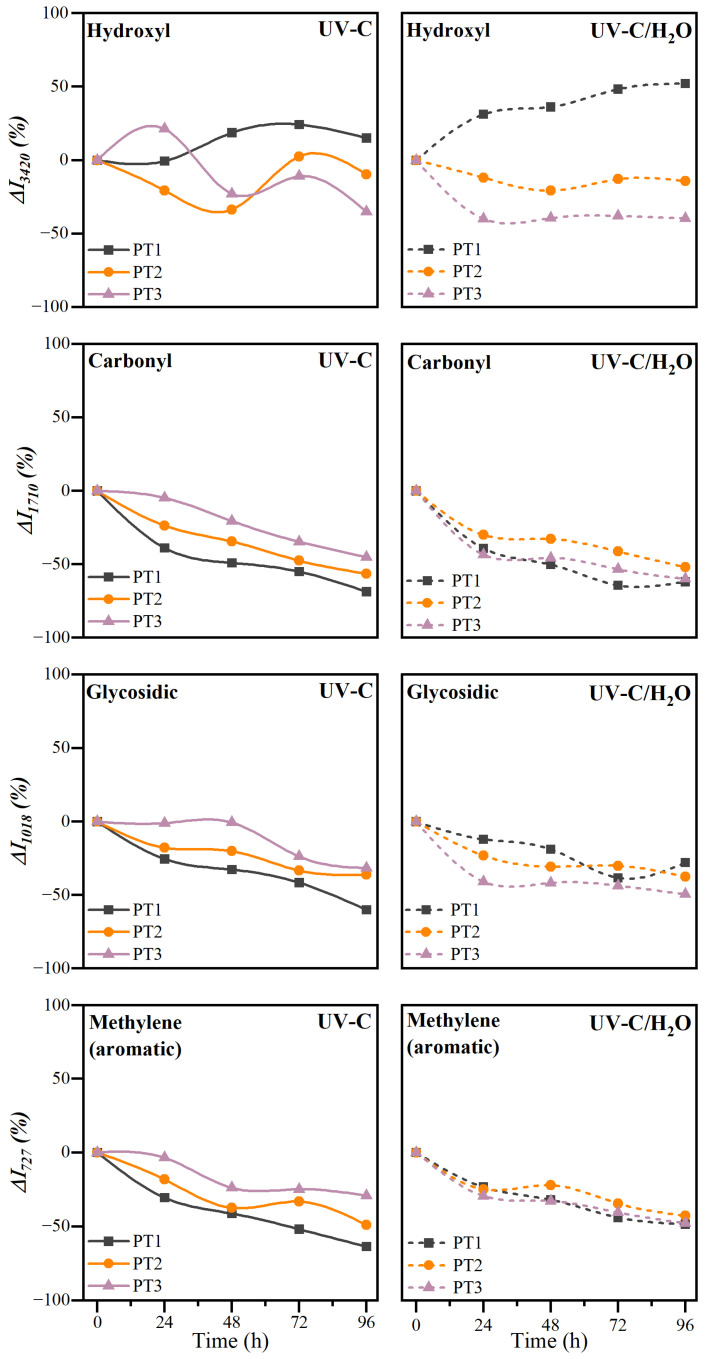
Intensity variation indexes for the hydroxyl (Δ*I*_3420_), carbonyl (Δ*I*_1710_), glycosidic (Δ*I*_1018_), and methylene bond (Δ*I*_727_) of PBAT/TPS films after UV-C irradiation in dry (UV-C) and during immersion (UV-C/H_2_O) conditions. Lines are given for the sake of visual aid.

In general, the absorbance spectra of the aged films show evidence that photodegradation reactions have occurred. Irradiation during dry and immersion conditions generated significant changes in the intensities associated with the functional groups of the PBAT/TPS films. These alterations may affect the signal’s intensity and cause shifts in the corresponding bands, indicating modifications in the composition and chemical structure. Among others, some observations include the following variations: in the specific IR bands at 3470 cm^−1^, related to the stretching of hydroxyl groups; at 1710 cm^−1^, associated with the C=O stretching of carboxylic acids; at 1018 cm^−1^, ascribed to the glycosidic bonds of starch molecules; and at 727 cm^−1^, ascribed to the out-of-plane bending vibration of the C–H in the phenylene ring in PBAT. Therefore, for a comprehensive evaluation, different indexes, namely Δ*I*_3420_, Δ*I*_1710_, Δ*I*_1018_, and Δ*I*_727_, were calculated as indicators of degradation. Thus, a more specific study of characteristic functional groups is presented in Figure 6.

Due to the chromophore nature of carbonyl groups in the ester linkages, and thus their susceptibility to photodegradation [10], variations in the signal at 1710 cm^−1^ were observed. Particularly, the intensity of the band progressively decreased, as shown by the Δ*I_1710_* index, and moved towards lower wavelength values. When UV-C irradiation was applied in dry conditions, this performance may be associated with the ester group breakage, where the irradiation directly impacts exposed films in the absence of water. The high energy of UV-C radiation may break ester groups of PBAT and provoke the generation of carboxyl units through the photodegradation reaction of Norrish Type I [41]. The hydrolysis of ester bonds during irradiation in water immersion may also contribute to the reduction of the ester carbonyls, and the generation of carboxylic acids [42]. This was corroborated with a greater displacement of the band towards lower wavelengths, and a more intense decrease in the initial stages of irradiation during immersion.

Additionally, the C–O groups in the ester bond may be also affected by the irradiation. The bands in the wavelength range between 1050 and 1300 cm^−1^ are ascribed to the stretching vibrations of the C–O bond [70]. However, ascribing the nature of this carbonyl group either to a carboxylic acid or to ester linkages may be challenging. In all the cases, both peaks showed less intense signals for greater treatment times, regardless of the UV-C irradiation in dry conditions or during water immersion. Nevertheless, it may be emphasized that whereas the higher wavelength signal of the C–O group remains larger than that at lower wavelengths during UV-C irradiation in dry conditions, a reverse performance was observed when irradiation was carried out during water immersion. These findings suggest that the high energy associated with UV-C radiation may disrupt the ester linkage of the PBAT during photodegradation in dry conditions [47], resulting in the formation of polymer chains with lower molecular weight [39]. Moreover, the above-mentioned performance during water immersion may demonstrate the contribution of water molecules, and the feasible occurrence of hydrolytic degradation of PBAT to a certain extent.

The ester bond breakage of PBAT is usually accompanied by a change in the vibrational signals linked to free, inter-, and intramolecular bonded hydroxyl groups (O–H) [71]. The intensity of this band at 3420 cm^−1^ was assessed through the Δ*I*_3420_ index. As occurred with the other indicators, a different behavior was found depending on the irradiation conditions and the composition of the films. In a dry irradiation environment, the intensity of the signal fluctuates over time, making it difficult to ascertain a tendency or pattern. In this case, UV-C light in the presence of oxygen facilitates oxidative reactions [87]. This often introduces new carbonyl groups in the form of aldehydes, ketones, and carboxylic acids, together with hydroxylation processes [88], that may promote the observed heterogeneous performance of the Δ*I*_3420_ index [42]. Furthermore, the occurrence of the Norrish II reaction must be considered [39,88], which is closely related to the photo-oxidation degradation. In the Norrish II reaction, UV absorption by carbonyl groups leads to the formation of terminal vinyl groups and chain scission, further contributing to the complex degradation process and the observed fluctuations in the hydroxyl group signals. Nevertheless, when UV-C irradiation was applied during water immersion, an interesting pattern was observed. In this case, for a low percentage of TPS, the balance leans toward an overall increase in hydroxyl groups because of the generation of new hydroxyls from the hydrolytic degradation of ester, despite the feasible release of TPS fraction. However, in compositions with greater TPS percentages, compatibility concerns, together with the reasonable phase separation, may result in the leaching of the hydrophilic TPS during immersion. This behavior was already suggested in previous sections due to the aggravated surface deterioration observed at the microscopic level when UV-C irradiation was applied during water immersion. Altogether, the loss of TPS domains seems to offset the increase in hydroxyl groups generated during hydrolysis, resulting in a net decrease in Δ*I_3420_* [89].

Subsequently, the reduction in the peaks in the 2850–2920 cm^−1^ range and at 727 cm^−1^ suggested a change in methylene (-CH_2_-) groups of the aliphatic hydrocarbon chains, and affectation of the aromatic out-of-plane C-H bending vibrations, respectively. These variations can be attributed primarily to chain scission caused by photo-oxidation both in the linear and aromatic units of PBAT, showing pronounced polymer damage induced by UV-C radiation [15]. This pattern seems to be slightly more severe during UV-C irradiation in dry conditions than in water immersion. In this case, water has attenuated photodegradation damage of aliphatic and aromatic hydrocarbon units, especially in films with higher PBAT proportions [83]. The absorption and attenuation of UV radiation by water provide a protective effect for the aliphatic and aromatic segments in the evaluated polymer films.

Finally, the reduction in the signal at 1018 cm^−1^ of the glycosidic bond (C–O–C) of the TPS domains given by the Δ*I*_1018_ index has been consistently observed across all formulations and irradiation conditions. On the one hand, during irradiation in dry conditions, the reduction of the glycosidic signal due to TPS may be mainly related to chain scission and radical generation on the glycosidic ring [90], in line with the previous observations in the microscopic appearance of the surface of the films. On the other hand, during irradiation in combination with water immersion, the previously observed changes in the hydrophilic performance, together with the generation of a porous and seriously damaged surface and the detected behavior of the hydroxyl band, may corroborate the dissolution of TPS domains that are being released into the aqueous media.

#### 3.2.4. Thermal Properties and Stability

As a consequence of both photodegradation and hydrolysis, the thermal properties and stability of PBAT/TPS blends can undergo considerable changes. To assess these alterations, detailed calorimetric and thermogravimetric analyses were conducted, providing crucial insights into how the crystalline structure, composition, and overall material performance evolve after exposure to UV-C irradiation and water immersion [91].

**Figure 7:**
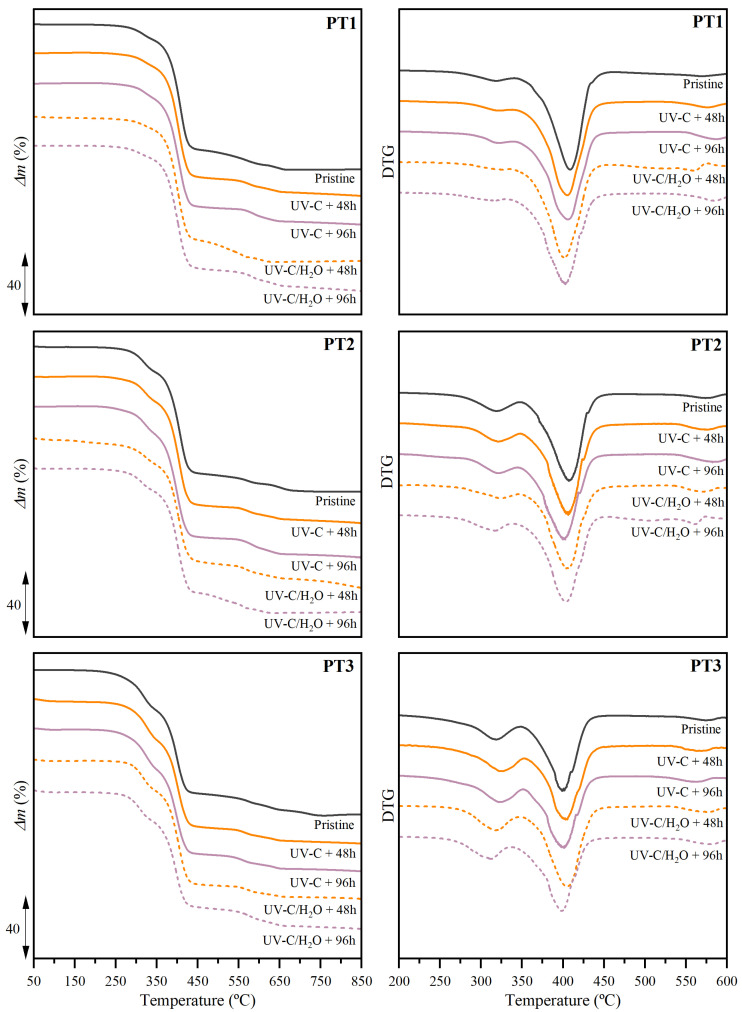
Thermogravimetric (TG) and first derivative thermogravimetric (DTG) curves obtained under an inert atmosphere for PBAT/TPS films after UV-C irradiation in dry (UV-C) and during immersion (UV-C/H_2_O) conditions.

First, the thermal stability was evaluated through thermogravimetric analyses. The obtained thermogravimetric (TG) and derivative thermogravimetric (DTG) curves for the different compositions and irradiation conditions are shown in Figure 7. The thermal stability was characterized in terms of the temperature for the 5% mass loss (*T_5%_*), the mass variation (Δ*m*), the peak temperatures (*T_d_*) for the TPS and PBAT fractions, and the solid residue percentage at 900 °C (*r*), the obtained values of which are gathered in Table S2 of the Supplementary Materials.

It was found that the *T*_5%_ remained virtually unchanged, regardless of the irradiation conditions. However, the dissimilar UV-C irradiation circumstances under dry and water- immersion conditions promoted different consequences regarding the thermal stability of the main decomposition process of the TPS and PBAT fractions.

When UV-C irradiation was applied under dry conditions, non-significant changes could be appreciated in their thermal stability performance. Both mass and temperatures remained virtually constant for the decomposition of the TPS and PBAT fractions. Only a small shoulder was observed in the PBAT main degradation process at slightly lower temperatures than the peak. These additional thermal events indicate chain scission, likely caused by UV-C treatment. This effect suggests the formation of low molecular weight fragments, which decompose at lower temperatures than the PBAT main degradation process.

However, when irradiation was carried out during water-immersion conditions, the mass loss changes were especially relevant for the contribution of the TPS fraction. The mass loss associated with the thermal decomposition of TPS decreased as a function of treatment time, and this performance was more pronounced in films with higher TPS content. Thus, compared to the untreated films, PT1, PT2, and PT3 showed a decrease of the TPS fraction of 13.5%, 25.1%, and 28.4%, respectively, after 96 h of treatment. The photodegradation along with the partial solubilization and release of the starch component reported in previous sections may motivate this performance. The microscopic porous structure found after being exposed to irradiation during immersion may serve as evidence that a portion of the TPS has been dissolved, which aligns with the mass loss reduction below 350 °C identified in the thermogravimetric traces. Nevertheless, non-perceivable changes were observed in the thermal decomposition behavior of the PBAT fraction in terms of peak temperatures. Only a greater mass loss percentage after 48 h of immersion could be appreciated, i.e., 2.7%, 3.4%, and 19.1% for PT1, PT2, and PT3, respectively, which is strictly due to the previously mentioned reduction of the contribution of the TPS fraction.

**Figure 8:**
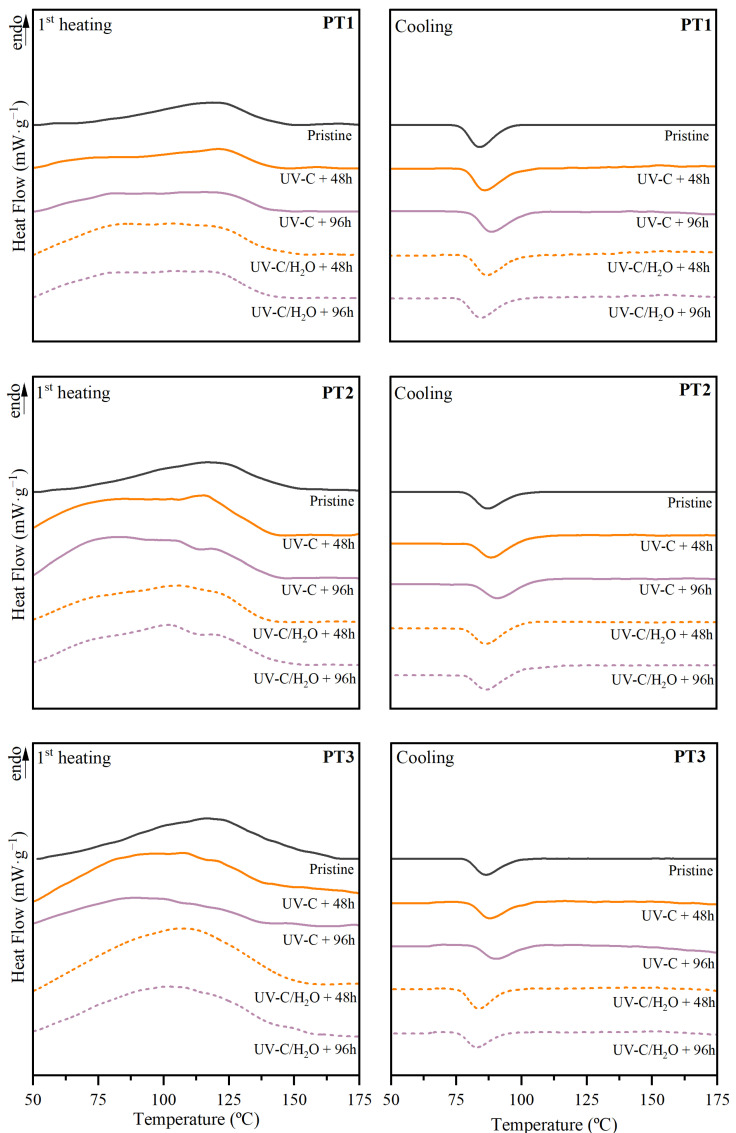
First heating and cooling scans of PBAT/TPS films after UV-C irradiation in dry (UV-C) and during immersion (UV-C/H_2_O) conditions.

**Figure 9:**
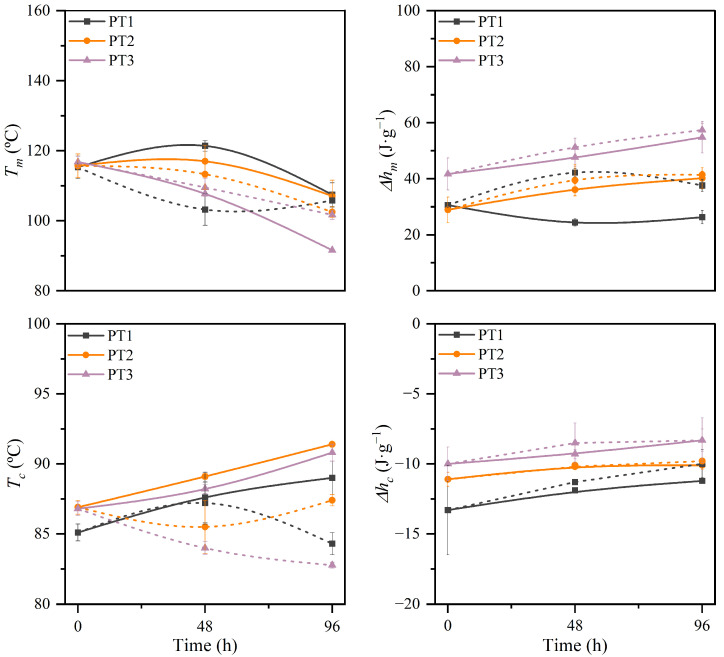
Variations of melting temperature (*T_m_*), melting enthalpy (Δ*h_m_*), crystallization temperature (*T_c_*), and crystallization enthalpy (Δ*h_c_*), all of them referred to as the PBAT fraction, of PBAT/TPS films after UV-C irradiation in dry (solid lines) and during immersion (short dash lines) conditions. Lines are given for the sake of visual aid.

Thermal properties were further evaluated through calorimetric analyses, considering the first heating and cooling scans. Figure 8 shows the thermograms for UV-C irradiated films during 48 h and 96 h under both dry and water-immersion conditions. The values of the melting temperature (*T_m_*) and enthalpy (Δ*h_m_*), and crystallization temperature (*T_c_*) and enthalpy (Δ*h_c_*), all of them referred to as the PBAT fraction, are presented in Table S3 in the Supplementary Materials. In general, irradiation promoted a shift of the broad melting peak of PBAT toward lower temperatures, along with the appearance of a shoulder near 80 °C, whereas the crystallization event of the PBAT moved to higher temperatures for films irradiated under dry conditions and showed an inverse performance when irradiation was applied during water immersion. With the purpose of visually and quantitatively evaluating these changes, the variation of *T_m_*, Δ*h_m_*, *T_c_*, and Δ*h_c_* is shown in Figure 9 as a function of irradiation time.

Even though the study of the degree of crystallinity (*X_c_*) comes up as one of the most important and widely used indicators for monitoring the degradation of polymers [26,30], the dissimilar mass loss perceived for the different compositions and immersion conditions due to the above-determined leaching of TPS fraction, and the remnant weight mass fraction of PBAT in the blends, could not be accurately estimated, and thus the study of the crystallinity degree was avoided in this section. However, to better understand the resulting crystallinity structure, the maximum lamellar thickness (*l_c_*) was calculated using the melting peak temperature.

Under UV-C irradiation in dry conditions, a significant decrease in the melting temperature was observed after 96 h with a maximum reduction of 22% and a slight increase in the crystallization temperature, about 5%. This performance involved a decrease in lamellar thickness of the PBAT crystalline structure from 11.0 nm for pristine materials to 9.0, 8.9, and 6.8 nm for PT1, PT2, and PT3 respectively. This reduction can be related to the presence of less perfect crystals for greater irradiation times [17]. Photodegradation can attack crystalline regions of PBAT, reducing the lamellar thickness by promoting chain scission [92]. As the chains are cleaved, particularly at the crystalline-amorphous interfaces, the molar mass may decrease, limiting the ability of polymer chains to fold and form stable lamellae. This process may disrupt the crystalline phase, causing thinner lamellae to form or even resulting in partial melting of crystalline structures at lower temperatures. Nevertheless, the melting enthalpy slightly increased, especially as the TPS percentage increased, indicating that the short chains generated during photodegradation may have restructured into new crystalline domains, which require more energy to melt. The increase in melting enthalpy highlights the complex interplay between degradation and recrystallization processes, where the generation of the additional crystalline domains compensates, to some extent, the deterioration of the original crystalline region.

A similar performance was observed after 96 h of UV-C irradiation in water-immersion conditions. Quantitatively, the melting temperature exhibits a reduction of 8.2%, 11.4%, and 12.9% for PT1, PT2, and PT3, respectively, and consequently, it results in lower lamellar thickness, with values of 8.7, 8.2, and 8.1 nm for PT1, PT2, and PT3, respectively, and so results in less perfect PBAT crystalline regions, suggesting the bulk deterioration of the films [93]. Higher melting enthalpy was found as a function of time, which could be correlated to the generation of more crystals of less quality during the applied treatment. During immersion, the amorphous region of PBAT mainly formed by BA segments can be preferentially degraded as a result of the hydrolysis in pH-neutral conditions [60]. Also, water could act as a plasticizing agent and therefore be a driving force for the reorganization of degraded polymer chains to organize into new crystalline domains, contributing to the increase in the Δ*h_m_*. Nevertheless, it is of high relevance considering the change in the composition of the sample as a function of time. Since the dissolution and lixiviation of the TPS fraction have been suggested during irradiation in water immersion, the composition of the sample in terms of PBAT and TPS percentages may vary. This behavior results in the increase of the weight percentage of PBAT in the sample, which can be correlated to the perceived greater melting enthalpy.

#### 3.2.5. Molar Mass

Molar mass analysis provides essential insights into the structural integrity and degradation extent of polymers after exposure to degrading conditions. Therefore, the molar mass distributions after UV-C irradiation during dry and water-immersion conditions were assessed using gel permeation chromatography. The acquired distributions were characterized in terms of the average molar mass in number (*M_n_*), the average molar mass in weight (*M_w_*), and the polydispersity index (*PDI*), all of them referred to the PBAT phase, the obtained values off which are gathered in Table 3.

In general, chain scission of PBAT was evident during UV-C irradiation in dry and water-immersion conditions. The overall performance involved a decrease in the values of *M_n_* and *M_w_*, together with an increase in the *PDI*. This pattern has been reported before for PBAT, where chain scission caused by photo-oxidation and photodegradation reactions resulted in a reduction in molecular weight [33,42,64].

Degradation in terms of molar mass decrease of PBAT was more severe during UV-C irradiation in the dry environment, where predominantly photo-oxidation reactions occurred. Quantitatively, after 96 h of irradiation in dry conditions, a reduction in *M_n_* of 26.9, 33.4, and 34.8% was found for PT1, PT2, and PT3, respectively, whereas it decreased about 6.9, 13.5, and 24.4% for UV-C irradiation during water immersion, respectively. Previous studies report that the absorption of UV-C radiation by water can lead to the formation of hydroxide (H-O^−^) and hydronium (H_3_O⁺) ions in the solution. The hydroxide ions are highly reactive and can initiate oxidative degradation [39], as well as ester hydrolysis, especially in alkaline conditions [25,60], while the hydronium ions can catalyze hydrolytic reactions [42], accelerating the aging of the material. However, in the current study, water can be considered a protective agent against photodegradation, given the lower overall chain scission observed during immersion. On the one hand, water molecules may exhibit relatively weak nucleophilic behavior in pH-neutral conditions, making it difficult for them to attack and hydrolyze the ester bonds [60]. Moreover, as described before, water absorbs UV-C radiation, which in turn reduces the dose received by the polymer film up to 97%. This behavior, due to the diffuse attenuation coefficient of water, corroborates the protective effect of water for the PBAT fraction versus UV-C irradiation [83], with main changes observed in immersed conditions primarily driven by water effects rather than UV-C contribution.

In terms of composition, degradation was accentuated for films with a higher percentage of TPS, regardless of the irradiation conditions. Under UV-C irradiation in dry conditions, chain scission in the PBAT phase was higher with greater TPS content due to the enhanced presence of hydrophilic hydroxyl groups from starch and plasticizers. These hydroxyl groups can accelerate photo-oxidative reactions when exposed to UV-C light, leading to faster degradation. Furthermore, TPS attracts moisture from the environment, which can increase local hydrolytic and oxidative reactions in the vicinity of PBAT [94]. This has been reported also for TPS/PLA blends under abiotic and biotic degradation [95]. Additionally, TPS often lacks UV stability, generating free radicals under UV-C exposure that may act as initiators of degradation and therefore accelerate chain scission in adjacent PBAT chains. This interplay between TPS and PBAT promotes a more extensive breakdown in the PBAT phase as the TPS fraction increases in the blend. When irradiation was applied during water immersion, greater TPS percentages also involved more degradation of PBAT after irradiation. However, a distinct mechanism should be proposed for this performance. More TPS in the blend formulation contributed to the formation of a deteriorated highly porous structure due to the partial dissolution of TPS, as previously observed in the surface morphology. This greater porosity expanded the contact surface with water, potentially enhancing the pathways for water penetration and hydrolytic chain breakage [96].

## 4. Conclusions

This study highlights the distinct abiotic degradation pathways of films of commercial PBAT/TPS blends under UV-C irradiation alone and in combination with water immersion. Also, the critical role of the TPS fraction was demonstrated, both increasing the susceptibility to photodegradation under dry conditions, and facilitating hydrolytic chain scission during water immersion, given the observed erosion as a consequence of TPS dissolution and leaching.

UV-C irradiation effectively disrupted the ester linkages within the PBAT phase due to photodegradation, initiating chain scission and significantly reducing its molar mass. Concurrently, water immersion created a porous structure by leaching the TPS fraction, thereby promoting further hydrolysis of PBAT. However, during water immersion, a more restrained impact on the molar mass was found, due to the diffuse attenuation coefficient of water. The combination of ultraviolet exposure and water immersion demonstrated a severe impact on the erosion and disintegration of PBAT/TPS blends, particularly in formulations with higher starch content. These findings highlight the susceptibility of these polymer blends to deterioration during water immersion, with structural and molecular alterations that compromise their global performance.

Overall, this work demonstrated the potential of applying strategies involving UV-C irradiation alone or in combination with exposure to water immersion to induce relevant physicochemical changes in PBAT/TPS films. By leveraging such advanced oxidation techniques, the degradation rates of bioplastics can be modulated, offering promising opportunities for their application in bioplastic lifecycle management.

## Figures and Tables

**Table 1:** Strategies to accelerate the biodegradation of biodegradable polymers.

Polymers	Valorization Strategy	Suggested Parameters	Key Indicators	Biodegradation Environment	Observation Reported	Ref.
PLA	UV irradiation + enzymatic degradation catalyzed with Proteinase K	UV-A light (***λ***: 300–700 nm) I: 25.5 mW·cm^−2^ T: 45 °C RH: 65% t: 60 h	Reduction of *M_n_*	Culture media T: 37 °C t: 10–60 h pH: 8–8.6	Accelerated depolymerization after 60 h of irradiation	[43]
PLA	UV irradiation + *Stenotrophomonas* *maltophilia LB 2–3*	UV-C light (***λ***: 185–245 nm) I: 6.41 × 10^−3^–3.22 mW·cm^−2^ t: 24 h	Reduction of *M_n_*, contact angle and mechanical properties	Compost T: 37 °C t: 24 h	Biodegradability increased after 8 h of UV-C irradiation but became more resistant with longer exposure times	[45]
Commercial PLA cups	UV irradiation + bioaugmentation + dairy wastewater sludge (*Pseudomonas geniculata WS3*)	UV-A-B-C light (***λ***: 340, 310 and 254 nm) t: 150 min T: room temperature	Significant reduction of *M_n_* after 2 h of irradiation	Soil T: 58 ± 2 °C RH: 40% pH: 4.3–7.9 Air flow: 25 mL·min^−1^	Enhanced PLA biodegradation with UV irradiation, along with the addition of dairy wastewater sludge and *P. geniculate WS3*	[44]
Cassava Starch	Ozone treatment + blending (PVA/NR) + biodegradation	T: 50 °C t: 50 min pH: 7 Ozone gas concentration: 20 mg L^−1^	Decreased crystallinity and swelling ratio in toluene and aqueous medium	Soil T: 27–28 °C RH: 85% pH: 7	Biodegradation improved with increasing Modified CS content (100% in 30 days with ≥ 15% MCS)	[46]
PBAT	UV irradiation + biodegradation	UV-A light (***λ***: 320–400 nm) t: 336 h I: 1.40 W·m^−2^·nm^−1^	Higher opacity and yellowish colour, decrease in TS and ε, higher brittleness, increase in *E*, reduction in *M_w_*, crosslinking	Compost t: 45 days	Photodegradation enhanced mineralization, only in the first stages, before crosslinking occurred after advanced irradiation	[10]

**Table 2:** Physico-chemical properties of pristine films of PBAT/TPS commercial blends.

		PT1	PT2	PT3
Thickness (µm)		172 ± 20	155 ± 10	123 ± 10
Macroscopic appearance		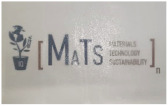	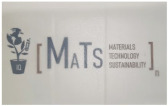	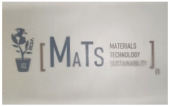
Color parameters	L*	85.7 ± 0.0	88.4 ± 0.3	82.1 ± 1.9
	a*	−2.0 ± 0.0	−2.4 ± 0.2	−2.3 ± 0.1
	b*	0.9 ± 0.0	3.4 ± 0.0	4.46 ± 0.1
Microscopic surface		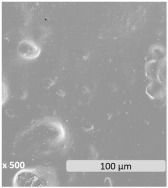	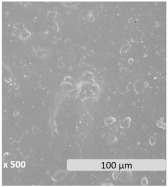	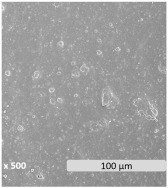
WCA (°)		79.1 ± 3.9	76.1 ± 2.7	68.6 ± 2.6
*T_m_* (°C)		121.4 ± 0.9	121.6 ± 3.4	119.9 ± 4.5
*T_d TPS_* (°C)		317.8 ± 0.2	317.4 ± 0.6	320.0 ± 1.5
*T_d PBAT_* (°C)		405.9 ± 4.5	403.9 ± 4.7	401.0 ± 2.3
*M_n_* (g·mol^−1^)		55,830	58,430	62,820
*M_w_* (g·mol^−1^)		113,450	119,750	118,610
*PDI*		2.03	2.05	1.89

**Table 3:** The molar mass of PBAT/TPS films in terms of average molar mass in number (*M_n_*), average molar mass in weight (*M_w_*), relative variations (Δ*M_n_,* Δ*M_w_*), and polydispersity index (*PDI*) after UV-C irradiation in dry (UV-C) and during immersion (UV-C/H_2_O) conditions.

		t (h)	*M_n_* (g·mol^−1^)	Δ*M_n_* (%)	*M_w_* (g·mol^−1^)	Δ*M_w_* (%)	*PDI*
PT1	-	0	55,830	-	113,450	-	2.03
	UV-C	96	40,830	−26.9	87,410	−23.0	2.14
	UV-C/H_2_O	96	51,980	−6.9	111,080	−2.1	2.14
PT2	-	0	58,430	-	115,750	-	1.98
	UV-C	96	38,910	−33.4	87,520	−24.4	2.25
	UV-C/H_2_O	96	50,570	−13.5	116,990	1.1	2.31
PT3	-	0	62,790	-	115,370	-	1.84
	UV-C	96	40,940	−34.8	96,370	−16.5	2.35
	UV-C/H_2_O	96	47,490	−24.4	100,650	−12.8	2.12

## Data Availability

The data supporting this study’s findings will be available in Zenodo (https://doi.org/10.5281/zenodo.15263461).

## Supplementary Materials

The following supporting information can be downloaded at: https://www.mdpi.com/article/10.3390/polym17091173/s1, Figure S1: Macroscopic changes of the PBAT/TPS films after UV-C irradiation in dry (UV-C) and during immersion (UV-C/H_2_O) conditions.; Table S1: Colour values obtained of PBAT/TPS films after UV-C irradiation in dry (UV-C) and during immersion (UV-C/H_2_O) conditions; Table S2: Characteristic degradation temperatures and mass loss percentages for the thermal decomposition of TPS and PBAT after UV-C irradiation in dry (UV-C) and during immersion (UV-C/H_2_O) conditions; Table S3: Thermal performance parameters of the PBAT fraction of PBAT/TPS after UV-C irradiation in dry (UV-C) and during immersion (UV-C/H_2_O) conditions.
