# Supplementary material for: UV-C and UV-C/H₂O-Induced Abiotic Degradation of Films of Commercial PBAT/TPS Blends
Source: Polymers (Basel). 2025 Apr 25;17(9):1173. doi: 10.3390/polym17091173 (PMC12073353; doi:10.3390/polym17091173)
Supplement: Supplementary file 1 [file polymers-17-01173-s001.zip › polymers-3526924-supplementary.pdf]

Supplementary Materials

UV-C and UV-C/H_2O-Induced Abiotic Degradation of Films of Commercial PBAT/TPS Blends

K. Gutiérrez-Silva [1], Antonio. J. Capezza [2], O. Gil-Castell [1,*], and J. D. Badia-Valiente [1,*]

[1] Research Group in Materials Technology and Sustainability (MATS), Department of Chemical Engineering, School of Engineering, University of Valencia, Av. Universitat s/n, 46100 Burjassot, Spain; karen.gutierrez@uv.es

[2] Fibre and Polymer Technology Department, KTH Royal Institute of Technology, Teknikringen 56, SE-100 44 Stockholm, Sweden; ajcv@kth.se

* Correspondence: oscar.gil@uv.es (O.G.-C.); jose.badia@uv.es (J.D.B.-V.)

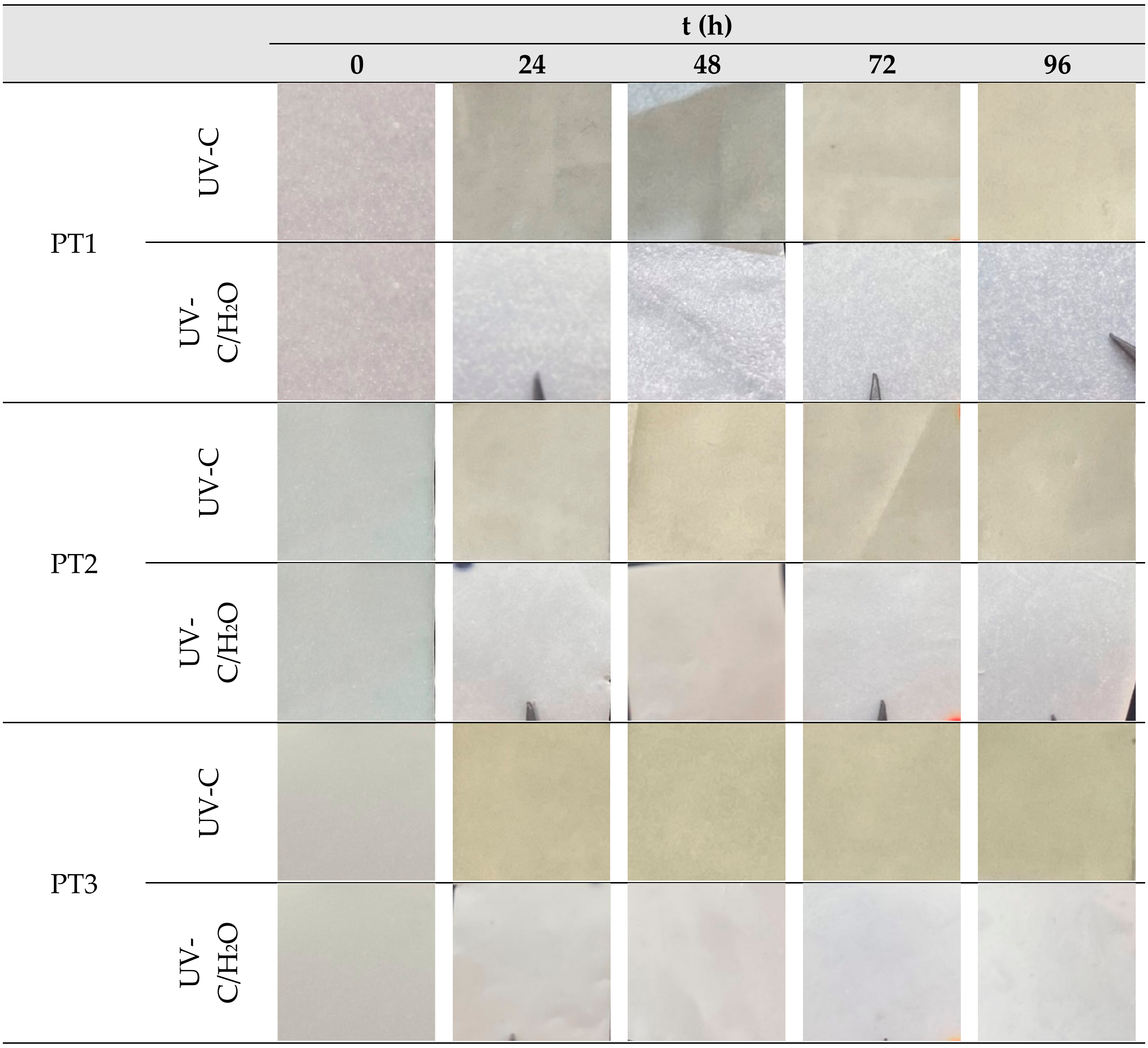

Figure S1. Macroscopic changes of the PBAT/TPS films after UV-C irradiation in dry (UV-C) and during immersion (UV-C/H_2O) conditions.

Table S1. Color values obtained of PBAT/TPS films after UV-C irradiation in dry (UV-C) and during immersion (UV-C/H_2O) conditions.

		t (h)	*L**	*a**	*b**	*ΔE*
PT1	UV-C	0	85.69 ± 0.03	-1.97 ± 0.03	0.91 ± 0.04	-
		24	82.94 ± 0.78	-2.43 ± 0.21	0.84 ± 0.02	2.79 ± 0.77
		48	83.38 ± 2.12	-2.00 ± 0.89	1.55 ± 0.02	2.40 ± 2.26
		72	82.49 ± 2.48	-2.46 ± 0.05	2.23 ± 0.09	3.50 ± 2.45
		96	83.01 ± 0.36	-4.09 ± 0.22	4.42 ± 0.38	4.90 ± 0.51
	UV-C/H_2O	0	85.69 ± 0.03	-1.97 ± 0.03	0.91 ± 0.04	-
		24	92.76 ± 0.17	-3.72 ± 0.06	-1.25 ± 0.09	7.60 ± 0.15
		48	91.74 ± 0.63	-4.23 ± 0.26	-1.21 ± 0.07	6.80 ± 0.64
		72	86.71 ± 0.48	-6.70 ± 0.22	-2.31 ± 0.08	5.81 ± 0.49
		96	84.66 ± 1.13	-7.95 ± 0.38	-2.59 ± 0.12	7.00 ± 1.16
PT2	UV-C	0	88.42 ± 0.31	-2.35 ± 0.24	3.38 ± 0.04	-
		24	81.87 ± 1.84	-2.66 ± 0.2	1.36 ± 0.04	6.86 ± 1.53
		48	81.41 ± 3.24	-3.33 ± 0.25	1.93 ± 0.13	7.23 ± 2.94
		72	80.86 ± 0.79	-4.00 ± 0.10	3.40 ± 0.19	7.74 ± 0.53
		96	77.29 ± 2.81	-4.37 ± 0.10	4.64 ± 0.14	11.38 ± 2.51
	UV-C/H_2O	0	88.42 ± 0.31	-2.35 ± 0.24	3.38 ± 0.04	-
		24	86.83 ± 2.34	-6.85 ± 1.08	-2.58 ± 0.86	7.64 ± 2.34
		48	89.35 ± 0.47	-5.91 ± 0.18	-1.74 ± 0.02	6.31 ± 0.17
		72	90.86 ± 0.37	-5.17 ± 0.17	-1.26 ± 0.10	5.96 ± 0.12
		96	90.44 ± 1.11	-5.51 ± 0.36	-1.08 ± 0.17	5.83 ± 0.83
PT3	UV-C	0	82.09 ± 1.88	-2.25 ± 0.06	4.46 ± 0.10	-
		24	88.85 ± 0.97	-3.18 ± 0.08	5.44 ± 0.33	6.89 ± 0.94
		48	86.79 ± 0.98	-3.21 ± 0.03	5.25 ± 0.18	4.86 ± 0.90
		72	84.99 ± 0.66	-3.51 ± 0.12	6.61 ± 0.31	3.82 ± 1.24
		96	82.44 ± 2.79	-3.99 ± 0.05	7.46 ± 0.27	3.48 ± 0.93
	UV-C/H_2O	0	82.09 ± 1.88	-2.25 ± 0.06	4.46 ± 0.10	-
		24	92.61 ± 0.24	-4.80 ± 0.04	0.35 ± 0.12	11.58 ± 1.64
		48	85.87 ± 0.37	-7.59 ± 0.19	-1.84 ± 0.25	9.09 ± 1.51
		72	87.16 ± 0.54	-7.21 ± 0.19	-1.85 ± 0.24	9.50 ± 1.35
		96	90.66 ± 0.84	-5.80 ± 0.33	-0.83 ± 0.32	10.68 ± 1.09

Table S2. Characteristic degradation temperatures, mass loss percentages and residue for the thermal decomposition of TPS and PBAT after UV-C irradiation in dry (UV-C) and during immersion (UV-C/H_2O) conditions.

		t (h)	$T_{5\%}$ (ºC)	$T_{d\ TPS}$ (ºC)	Δm_{TPS} (%)	$T_{d\ PBAT}$ (ºC)	Δm_{PBAT} (%)	r (%)
PT1	UV-C	0	312.8 ± 6.1	317.8 ± 0.2	9.9 ± 0.5	405.9 ± 4.5	73.6 ± 1.2	4.6 ± 0.5
		48	318.3 ± 7.5	320.6 ± 2.0	8.9 ± 0.8	400.9 ± 5.3	71.7 ± 4.1	3.6 ± 2.0
		96	320.7 ± 2.8	320.3 ±0.9	9.7 ± 0.7	405.7 ± 0.1	73.4 ± 0.4	6.6 ± 2.5
	UV-C/ H_2O	0	312.8 ± 6.1	317.8 ± 0.2	9.9 ± 0.5	405.9 ± 4.5	73.6 ± 1.2	4.6 ± 0.5
		48	309.9 ± 0.6	320.8 ± 1.9	8.1 ± 1.4	399.7 ± 7.0	75.6 ± 2.6	2.6 ±1.6
		96	314.4 ± 1.0	313.7 ± 2.6	7.5 ± 0.1	401.5 ± 2.2	74.2 ± 1.4	8.1 ± 4.4
PT2	UV-C	0	301.2 ± 4.1	318.6 ± 1.1	16.6 ± 0.2	403.9 ± 4.7	64.3 ± 3.7	4.4 ± 1.0
		48	302.4 ± 1.8	319.2 ± 1.7	16.1 ± 0.7	401.5 ± 6.9	66.2 ± 4.1	1.8 ± 2.5
		96	299.5 ± 1.9	322.1 ± 0.2	17.5 ± 0.4	404.6 ± 2.5	67.7 ± 1.8	2.8 ± 2.1
	UV-C/ H_2O	0	301.2 ± 4.1	318.6 ± 1.1	16.6 ± 0.2	403.9 ± 4.7	64.3 ± 3.7	4.4 ± 1.0
		48	290.3 ± 1.6	324.1 ± 1.1	14.7 ± 1.8	404.0 ± 3.4	66.5 ± 0.6	0.2 ± 0.3
		96	297.3 ± 1.3	315.1 ± 1.7	14.3 ± 0.4	403.3 ± 0.5	67.7 ± 0.6	5.8 ± 0.9
PT3	UV-C	0	281.8 ± 3.0	319.8 ± 1.3	25.5 ± 0.6	401.0 ± 2.3	55.2 ± 0.1	4.8 ± 0.8
		48	279.4 ± 8.1	322.9 ± 1.6	25.7 ± 0.4	401.4 ± 1.8	57.6 ± 0.3	6.0 ± 1.7
		96	282.1 ± 0.3	322.1 ± 0.4	26.3 ± 0.2	401.3 ± 0.5	56.9 ± 1.4	5.0 ± 2.3
	UV-C/ H_2O	0	281.8 ± 3.0	319.8 ± 1.3	25.5 ± 0.6	401.0 ± 2.3	55.2 ± 0.1	4.8 ± 0.8
		48	295.5 ± 2.8	319.5 ± 1.6	20.5 ± 1.5	403.2 ± 2.1	65.7 ± 4.6	4.3 ± 3.1
		96	288.0 ± 5.1	310.9 ± 1.4	18.2 ± 0.1	399.9 ± 0.7	64.2 ± 1.6	4.7 ± 1.0

Table S3. Thermal performance parameters (temperatures, enthalpies and lamellar thickness) of the PBAT fraction of PBAT/TPS after UV-C irradiation in dry (UV-C) and during immersion (UV-C/H_2O) conditions.

		t (h)	T_m (ºC)	Δh_m (J/g)	T_c (ºC)	Δh_c (J/g)	l_c (nm)
PT1	UV-C	0	115.3 ± 3.2	30.6 ± 0.7	85.1 ± 0.6	-13.3 ± 3.2	10.6 ± 2.9
		48	121.4 ± 1.5	24.4 ± 1.3	87.6 ± 1.8	-11.9 ± 0.1	12.1 ± 2.9
		96	107.4 ± 3.4	26.3 ± 2.4	89.0 ± 1.2	-11.2 ± 0.2	9.0 ± 2.9
	UV-C/ H_2O	0	115.3 ± 3.2	30.6 ± 0.7	85.1 ± 0.6	-13.3 ± 3.2	10.6 ± 2.9
		48	103.5 ± 4.5	42.2 ± 1.5	87.2 ± 1.5	-11.3 ± 0.1	8.3 ± 2.9
		96	105.8 ± 1.9	37.6 ± 2.2	84.3 ± 0.8	-10.0 ± 1.0	8.7 ± 2.9
PT2	UV-C	0	115.7 ± 3.4	28.9 ± 4.6	86.9 ± 0.5	-11.1 ± 0.5	10.7 ± 2.9
		48	117.0 ± 4.6	36.1 ± 2.1	89.1 ± 0.2	-10.1 ± 0.2	11.1 ± 3.0
		96	107.1 ± 4.5	40.2 ± 3.8	91.4 ± 0.1	-10.1 ± 0.3	8.9 ± 3.0
	UV-C/ H_2O	0	115.7 ± 3.4	28.9 ± 4.6	86.9 ± 0.5	-11.1 ± 0.5	10.7 ± 2.9
		48	113.3 ± 4.4	39.5 ± 5.6	85.5 ± 1.9	-10.2 ± 0.2	10.2 ± 3.0
		96	102.5 ± 1.5	41.4 ± 1.3	87.4 ± 0.4	-9.8 ± 0.1	8.2 ± 2.9
PT3	UV-C	0	116.8 ± 1.6	41.7 ± 5.7	86.8 ± 0.5	-10.0 ± 1.2	11.0 ± 2.9
		48	107.7 ± 5.9	47.6 ± 3.1	88.2 ± 0.3	-9.3 ± 0.3	9.0 ± 3.0
		96	91.6 ± 6.1	54.8 ± 5.6	90.8 ± 0.6	-8.3 ± 0.8	6.8 ± 3.0
	UV-C/ H_2O	0	116.8 ± 1.6	41.7 ± 5.7	86.8 ± 0.5	-10.0 ± 1.2	11.0 ± 2.9
		48	109.5 ± 2.5	51.2 ± 3.3	84.0 ± 0.5	-8.5 ± 1.4	9.3 ± 2.9
		96	101.7 ± 1.3	57.4 ± 2.6	82.8 ± 0.2	-8.3 ± 1.6	8.1 ± 2.9
